# Aconitase Regulation of Erythropoiesis Correlates with a Novel Licensing Function in Erythropoietin-Induced ERK Signaling

**DOI:** 10.1371/journal.pone.0023850

**Published:** 2011-08-22

**Authors:** Anne-Laure Talbot, Grant C. Bullock, Lorrie L. Delehanty, Martin Sattler, Zhizhuang Joe Zhao, Adam N. Goldfarb

**Affiliations:** 1 Department of Pathology, University of Virginia School of Medicine, Charlottesville, Virginia, United States of America; 2 Department of Medical Oncology, Dana-Farber Cancer Institute, Boston, Massachusetts, United States of America; 3 Department of Pathology, University of Oklahoma Health Sciences Center, Oklahoma City, Oklahoma, United States of America; Yale Medical School, United States of America

## Abstract

**Background:**

Erythroid development requires the action of erythropoietin (EPO) on committed progenitors to match red cell output to demand. In this process, iron acts as a critical cofactor, with iron deficiency blunting EPO-responsiveness of erythroid progenitors. Aconitase enzymes have recently been identified as possible signal integration elements that couple erythropoiesis with iron availability. In the current study, a regulatory role for aconitase during erythropoiesis was ascertained using a direct inhibitory strategy.

**Methodology/Principal Findings:**

In C57BL/6 mice, infusion of an aconitase active-site inhibitor caused a hypoplastic anemia and suppressed responsiveness to hemolytic challenge. In a murine model of polycythemia vera, aconitase inhibition rapidly normalized red cell counts, but did not perturb other lineages. In primary erythroid progenitor cultures, aconitase inhibition impaired proliferation and maturation but had no effect on viability or ATP levels. This inhibition correlated with a blockade in EPO signal transmission specifically via ERK, with preservation of JAK2-STAT5 and Akt activation. Correspondingly, a physical interaction between ERK and mitochondrial aconitase was identified and found to be sensitive to aconitase inhibition.

**Conclusions/Significance:**

Direct aconitase inhibition interferes with erythropoiesis in vivo and in vitro, confirming a lineage-selective regulatory role involving its enzymatic activity. This inhibition spares metabolic function but impedes EPO-induced ERK signaling and disturbs a newly identified ERK-aconitase physical interaction. We propose a model in which aconitase functions as a licensing factor in ERK-dependent proliferation and differentiation, thereby providing a regulatory input for iron in EPO-dependent erythropoiesis. Directly targeting aconitase may provide an alternative to phlebotomy in the treatment of polycythemia vera.

## Introduction

Production of red blood cells, or erythropoiesis, is regulated by the cytokine erythropoietin (EPO) in conjunction with iron. In the context of an adequate iron supply, EPO promotes proliferation, differentiation, and survival of erythroid progenitors, beginning at the colony forming unit-erythroid (CFU-E) stage. Iron restriction, i.e. diminished amounts of bio-available iron, results in diminished red cell production due to decreased erythroid proliferation and maturation [1]. Iron regulation of erythropoiesis has been documented in many experimental models including rats, where iron deficiency causes a defect in the transition from the CFU-E stage to the proerythroblast stage [2]. Iron modulation of EPO bioactivity occurs clinically in patients whose response to recombinant EPO can be augmented by exogenous iron despite adequate iron stores [3]. Thus, iron sensing mechanisms in the erythroid compartment function in a rheostatic manner to adjust output based on iron availability.

Aconitase enzymes have recently been identified as mediators of the erythroid response to iron restriction [4]. In mammals, aconitases consist of mitochondrial and cytosolic isoenzymes that both utilize a prosthetic 4Fe-4S cubane iron-sulfur cluster group at their active site to interconvert the metabolites citrate and isocitrate [5]. They are highly sensitive to intracellular iron levels and redox conditions. Cellular iron deprivation causes loss of the α-Fe^2+^ group from the iron-sulfur cluster, while oxidative stress induces complete cluster disassembly, both of which conditions inactivate enzymatic function [6], [7]. An additional level of enzymatic regulation may arise from phosphorylation [8], [9]. Both isoforms also exert non-enzymatic functions through interaction with nucleic acid targets. Cytosolic aconitase, in its role as Iron Regulatory Protein 1 (IRP1), binds iron response elements within mRNA sequences of a cohort of iron responsive genes and regulates their expression [10]. Mitochondrial aconitase contributes to the protein complexes assembled with the mitochondrial genome and participates in mitochondrial DNA maintenance [11].

EPO engagement of its receptor activates the associated cytosolic tyrosine kinase JAK2, which in turn activates multiple signal transduction pathways critical in erythropoiesis. One such pathway consists of RAF-MEK-ERK [12]–[14]. In many cell types, ERK activation exerts both positive and negative effects on proliferation and differentiation, with signal output determined by kinetics and magnitude of activation, subcellular localization, scaffolds, and crosstalk with other signaling modules [15]. Known subcellular locations for ERK include the plasma membrane, endosomes, Golgi apparatus, nucleus, and mitochondria [16]–[20]. With regard to erythropoiesis, enforced ERK activation by mutant N-Ras V12 expression in ex vivo murine fetal liver erythroblast cultures blocked differentiation and promoted proliferation [21]. In vivo studies also suggest that ERK1 signaling negatively regulates red cell production. In these studies, ERK1 null mice displayed a higher basal rate of splenic erythropoiesis and responded more rapidly to hemolytic challenge [22]. On the other hand, MEK inhibition strongly diminished the yield of erythroid colonies from wild type murine marrow cultured in the presence of EPO [23]. Thus, as with many cell types, ERK signaling exerts both positive and negative effects during erythropoiesis.

Iron regulation of erythropoiesis appears to involve aconitase-mediated alterations in EPO signal transduction and cellular metabolism [4]. Because iron deprivation affects numerous cellular pathways in addition to aconitase, the current studies have employed a targeted inhibitory approach to more specifically define the role of aconitase activity in erythropoiesis. These studies, using a potent active-site inhibitor, revealed that aconitase activity regulates in vivo steady-state erythropoiesis, as well as responsiveness to acute anemia. Ex vivo studies confirmed this regulatory relationship, with aconitase inhibition affecting proliferation and differentiation but not viability. Surprisingly, doses of the aconitase inhibitor that effectively inhibited erythropoiesis had no effects on cellular ATP levels or on EPO induction of JAK2 activation but did block ERK-mediated phosphorylation of RSK substrates. A physical interaction between aconitase and ERK was observed and found to be sensitive to the inhibitor.

As a putative mediator of the erythroid response to iron restriction, aconitase activity could offer a target for controlling disease in patients with polycythemia vera (PV). PV is a hematopoietic stem cell disorder associated with an activating mutation in JAK2 [24], [25] that causes expansion of red cell mass and possible thrombotic and hemorrhagic complications [26]. The treatment of choice for normalizing red cell mass has consisted of induction of iron deficiency through repeated phlebotomy. In this study, low-dose infusion of the aconitase inhibitor rapidly normalized red cell counts in a murine model of PV. Thus the novel pathway for aconitase regulation of erythropoiesis described herein bears potential for therapeutic manipulation in the setting of oncogenic signaling.

## Results

### Induction of anemia by the aconitase inhibitor fluoroacetate

Aconitase enzymes have recently been implicated in the erythroid iron restriction response in ex vivo progenitor cultures and in iron deficient mice [4]. To define the contribution of aconitase enzymatic activity to erythropoiesis in vivo, the potent and specific active-site inhibitor of aconitase fluoroacetate (FA) was administered to mice [27]. Previous studies have shown that FA can be safely administered to mice at a dose of 4 mg/kg/day using a continuous infusion pump, a regimen that causes a 60% increase in resting serum citrate levels [28]. Accordingly, an infusion of FA or 0.9% saline was delivered to C57BL/6 mice at this dosing rate [28]. Complete blood counts obtained after 14 days revealed anemia in the FA-treated animals compared with controls (Table 1). In particular, red blood cell (RBC) counts, hemoglobin and hematocrit were significantly decreased in the FA-treated group. Importantly, the mean corpuscular hemoglobin concentration (MCHC) was unaffected by aconitase inhibition (Table 1), in contrast to the hypochromic anemias associated with defective heme biosynthesis (see Discussion). With regard to non-erythroid cells, FA treatment at 4 mg/kg/day caused a slight decrease in absolute neutrophil counts (ANC) and did not affect platelets (Table 1). The inhibition of the ANC was not reproducible in subsequent experiments. By contrast, the erythroid abnormalities associated with FA infusion were highly reproducible in multiple experiments; even those using a 2 mg/kg/day dosing regimen (see Table S1). To confirm that FA treatment inhibited aconitase activity in vivo, serum citrate levels were measured, and the 2 mg/kg/day dosing regimen was found to cause a 1.9-fold increase in mean serum citrate concentration (Fig. S1).

**Table 1:** Hematologic parameters of C57BL/6 mice treated for 14 days with saline (0.9%) or FA (4 mg/kg/day).

Blood parameter	Saline (n = 10)	FA-treated (n = 10)	*P* value
**RBC, ×10^6^/µL**	9.32±0.7	7.45±0.09	<0.001*
**Hematocrit, %**	44.0±3.5	36.0±4.4	<0.005*
**Hemoglobin, g/dL**	14.7±1.08	11.6±1.0	<0.001*
**MCV, fL**	47.24±0.71	48.49±0.7	0.01*
**MCHC, g/dL**	33.6±2.76	32.41±3.10	0.38
**Platelets, ×10^3^/µL**	804±84	734±7.3	0.067
**Neutrophils, ×10^3^/µL**	1.28±0.3	0.89±0.4	0.02*

RBC indicates Red Blood Cell; MCV, Mean Corpuscular Volume; MCHC, Mean Corpuscular Hemoglobin Concentration.

Data are presented as mean ± SD.

*significantly different from value for saline-treated mice.

**Figure 1:**
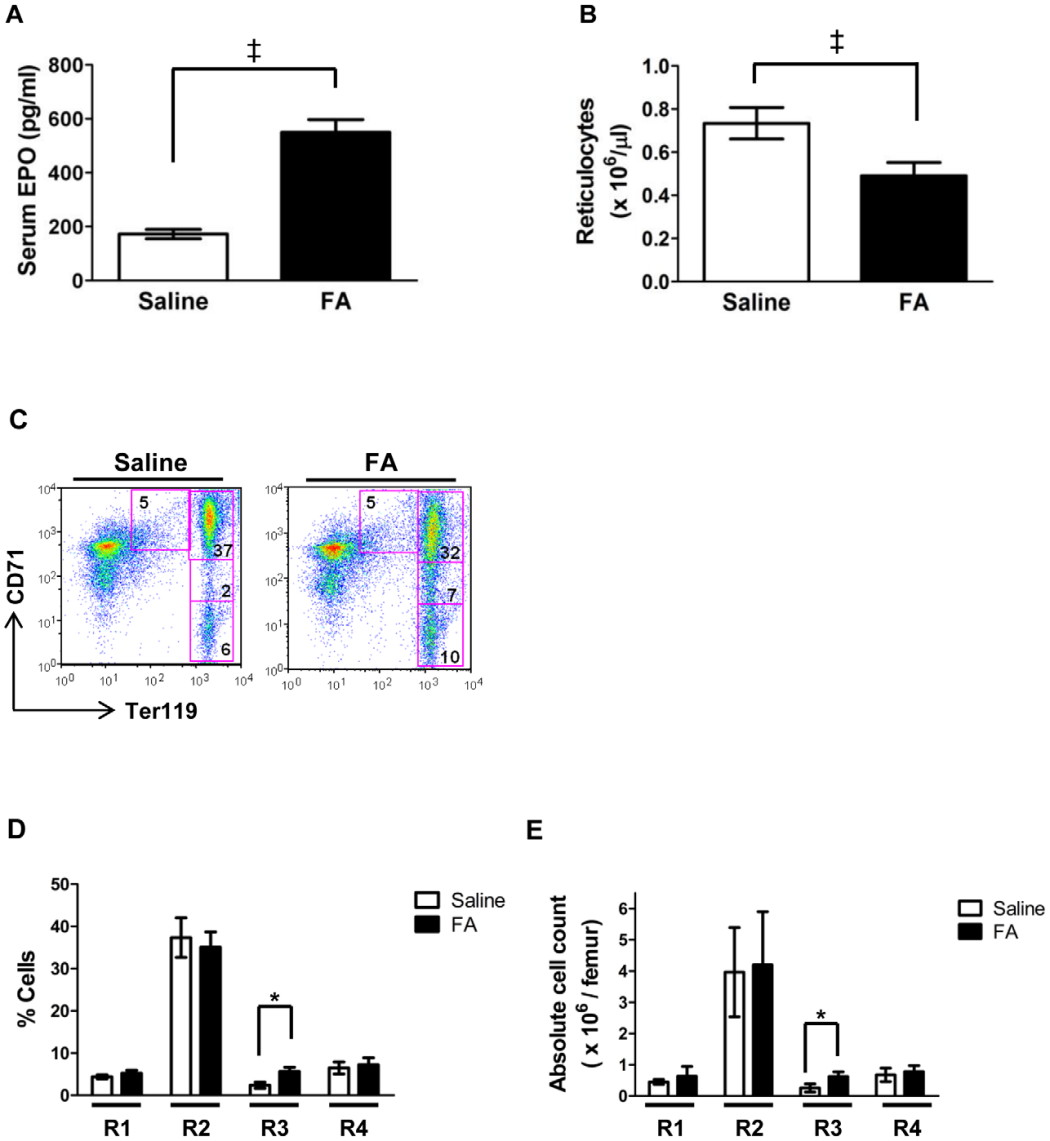
In vivo effects of aconitase inhibition. (**A**) Serum EPO levels in mice treated for 14 days with an infusion of saline (0.9%) or FA (4 mg/kg/day). Data represent mean ± SD; n = 9 for saline group and 10 for FA group; ^‡^ *P*<0.001. (**B**) Absolute reticulocyte counts in mice treated as in panel **A**. Data represent mean ± SD; n = 10 per group; ^‡^ *P*<0.001. (**C**) Erythroid maturation in marrows from mice who received a four-week infusion of FA (2 mg/kg/day) or saline (0.9%). Representative flow cytometry plots are shown. Boxes indicate erythroid developmental stages R1–R4, from top left (R1) to bottom right (R4). (**D**), (**E**) Summary of data from panel **C** displayed as cell percentages (**D**) or absolute cell numbers per femur (**E**) in regions R1–R4. Data is presented as mean ± SD; n = 4 per group; ^*^ *P*<0.05.

Further characterization revealed the anemia to be associated with a 2.5-fold increase in serum EPO levels (Fig. 1A) and a significant decrease in absolute reticulocyte counts (Fig. 1B). Flow cytometric analysis of bone marrow erythroid maturation employed co-staining for CD71 and Ter119, as has been described (Fig. 1C) [29]. The FA-treated mice showed a significant increase in frequency and absolute number of R3 erythroid precursors (CD71^Intermediate^ Ter119^Bright^) (Figs. 1C–E). This defect in erythroid maturation was observed in multiple independent experiments involving in vivo aconitase inhibition. Maturation was also analyzed using a recently described approach that resolves Ter119^+^ erythroid precursors into five distinct subpopulations based on CD44 levels and forward scatter (FSC) [30]. This approach showed a significant increase in the frequency of CD44^−^ FSC^Low^ cells (stage V) associated with FA treatment (Fig. S2).

### Aconitase inhibition impairs responsiveness to anemic challenge

**Figure 2:**
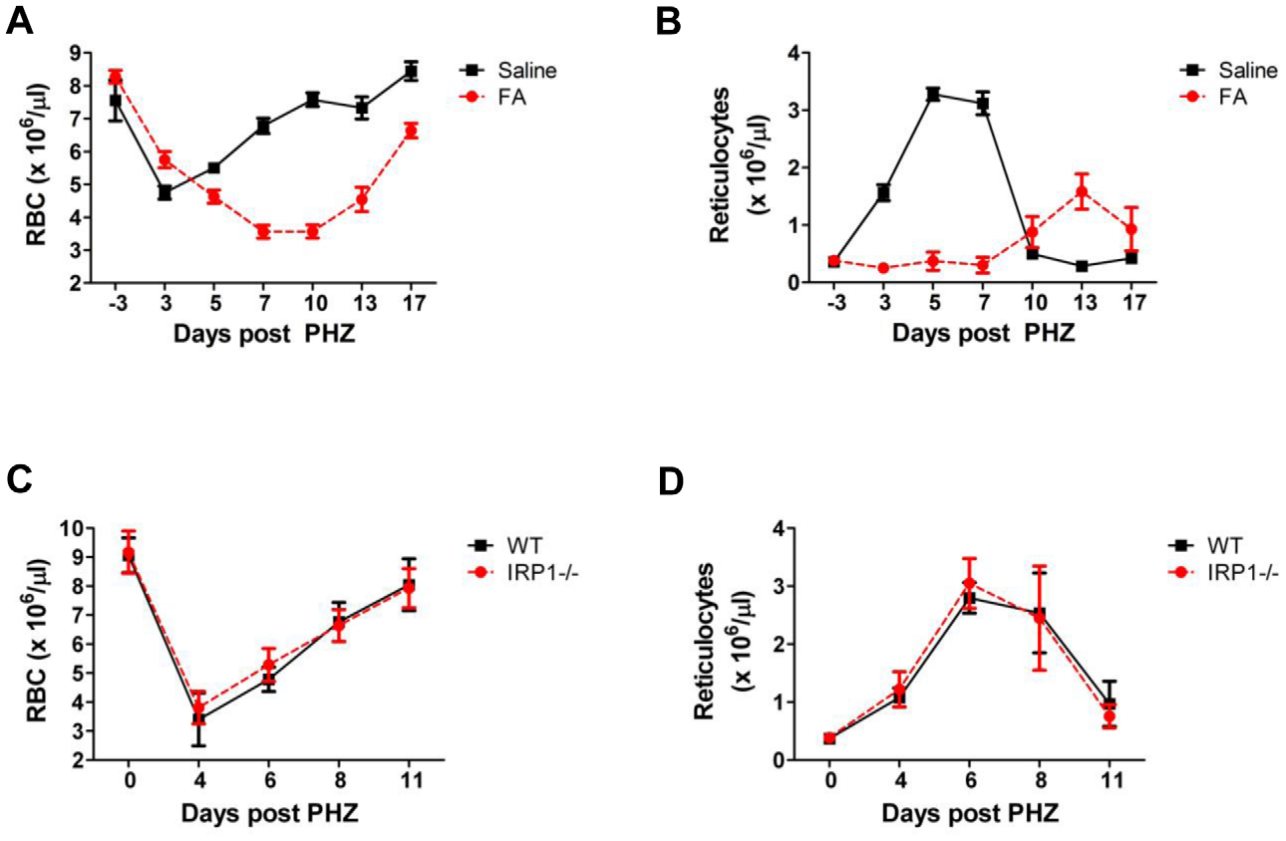
Inhibition of aconitase impairs responsiveness to anemic challenge. (**A**) RBC and (**B**) absolute reticulocyte counts in PHZ-challenged mice treated with either FA (4 mg/kg/day; n = 11) or saline (0.9%; n = 6). PHZ injections on days 0 and 1 consisted of 30 mg/kg/day for the FA group and 60 mg/kg/day for the saline group. Data represent mean ± SD. (**C**) RBC and (**D**) absolute reticulocyte counts in IRP1−/− and wild type (WT) mice. PHZ doses for both groups consisted of 60 mg/kg/day on days 0 and 1. Data represent mean ± SD; n = 8 per group.

Erythropoiesis can increase dramatically in response to acute anemia. To determine whether aconitase inhibition interferes with the capacity to respond to anemic challenge, mice treated with saline or 4 mg/kg/day FA received intraperitoneal injections of the hemolytic agent phenylhydrazine (PHZ) with dosage reduction made for the FA-treated group to prevent lethality (see Materials and Methods). PHZ treatment typically leads to an RBC nadir on day 3 post-injection followed by complete recovery on day 7 [31]. Despite receiving half the standard PHZ dose, mice treated with FA developed an anemia that was much more pronounced and prolonged than that of the control mice. Specifically, their RBC counts reached nadir on day 7 instead of day 3 and did not return to baseline even 17 days after PHZ administration (Fig. 2A). In addition, their reticulocyte response was delayed and diminished (Fig. 2B).

Further characterization of the role of aconitase in the response to acute anemia employed mice lacking cytosolic aconitase (IRP1−/− mice) [32]. These mice, along with wild-type aged-matched controls, received standard doses of PHZ and were monitored for responsiveness. As shown in Figs. 2C and 2D, IRP1−/− mice responded to PHZ-induced anemia in a manner indistinguishable from wild type mice. The starting RBC counts, the magnitude of their decrease, and the rate of recovery were not significantly different in the two groups; both also exhibited a similar reticulocyte response during the recovery phase. These results indicate that cytosolic aconitase is dispensable for both steady-state erythropoiesis and for responsiveness to acute anemia (see Discussion).

### Aconitase inhibition affects erythropoiesis in a cell-intrinsic manner

**Figure 3:**
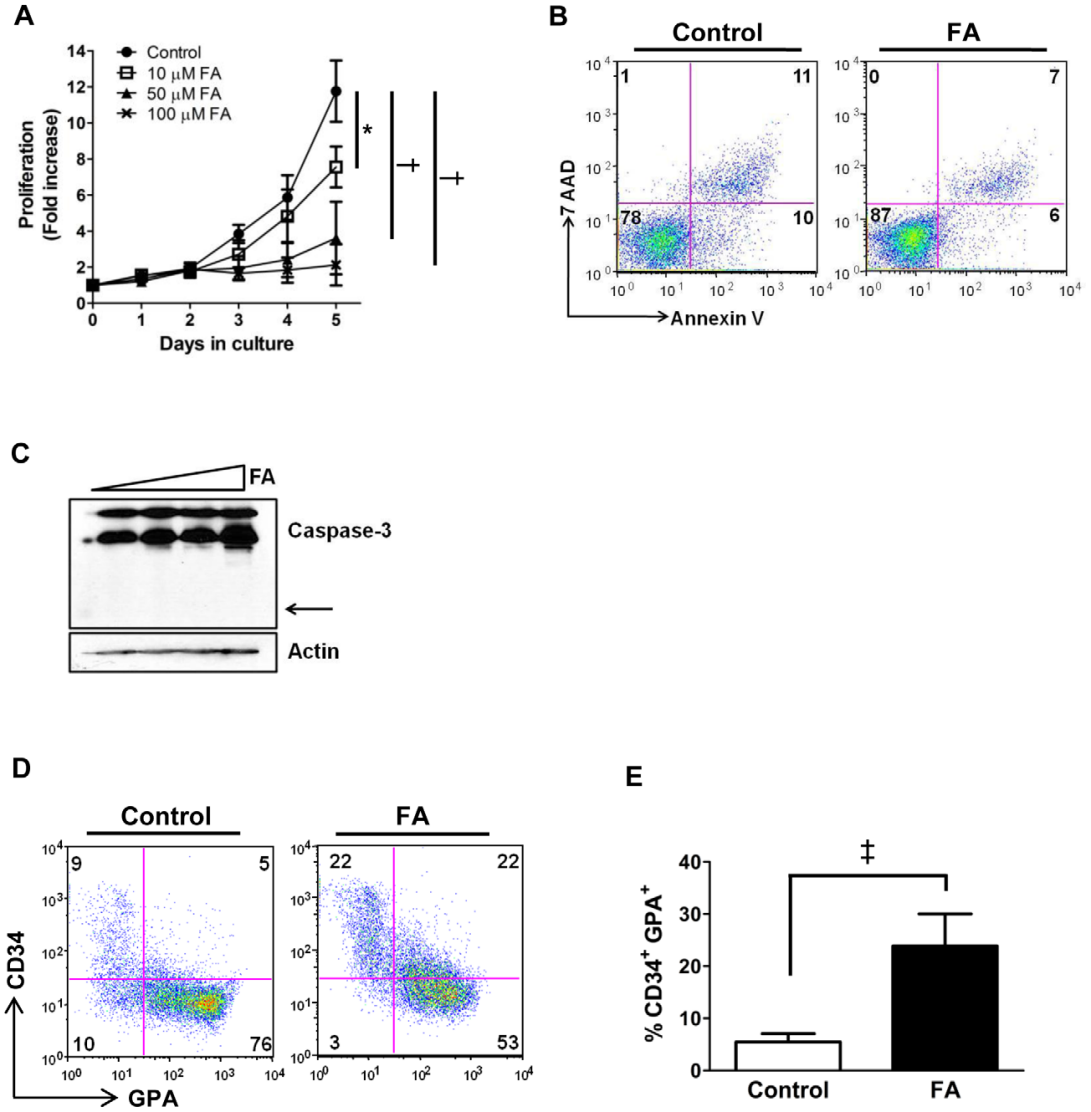
Effects of aconitase inhibition on erythroid proliferation, survival and maturation in vitro. (**A**) Fold increase in number of primary human progenitors cultured for five days in erythroid medium with the indicated doses of FA. Shown are mean ± SD for three independent experiments; ^*^ *P*<0.05; ^†^ *P*<0.01. (**B**) Survival of cells from day 5 erythroid cultures ±50 µM FA assessed by staining with 7-amino actinomycin D (7-AAD) and annexin V-PE followed by flow cytometry; numbers indicate percentages of cells in each quadrant. (**C**) Apoptotic signaling in cells from day 5 erythroid cultures with 0, 10, 50, and 100 µM FA assessed by immunoblotting of whole cell lysates for caspase-3 (arrow indicates position of cleavage product). (**D**) Differentiation of erythroid progenitors cultured as in panel **B**. Surface expression of CD34 and GPA was assessed by flow cytometry on live cells; numbers indicate percentages of cells in each quadrant. (**E**) Multiple independent experiments as in panel **D** show an increase in the percentage of CD34^+^ GPA^+^ cells associated with FA treatment. Shown are mean ± SD; n = 6; ^‡^ *P*<0.001.

The in vivo effects of FA infusion support a role for aconitase activity in the regulation of erythropoiesis but could also reflect indirect influences mediated by the marrow microenvironment. Therefore, ex vivo primary cultures were used to examine the effects of aconitase inhibition directly on erythropoiesis. In these experiments, purified human CD34^+^ cells were cultured in unilineage erythroid medium (see Materials and Methods) with or without fluoroacetate. Aconitase inhibition caused a dose-dependent decrease in proliferation over a five day culture period, with 10 µM causing a statistically significant decrease and 100 µM abrogating growth altogether (Fig. 3A). The proliferation defect was not secondary to cell death, as FA treatment had minimal effects on overall viability and no effects on apoptotic signaling (Figs. 3B,C). All subsequent experiments employed 50 µM FA because this dose reproducibly inhibited proliferation and maturation (see below) without affecting viability. In addition, this dose strongly inhibited aconitase activity in erythroid progenitors, causing a 10-fold increase in intracellular citrate levels (Fig. S3).

Erythroid differentiation was assessed by examining the expression of two developmental markers: CD34, a stem cell antigen rapidly downregulated after the early burst forming unit-erythroid (BFU-E) stage, and glycophorin A (GPA), an erythroid-specific antigen appearing initially at the proerythroblastic stage and continually increasing with differentiation [33], [34]. Addition of FA to the erythroid cultures impaired the downregulation of CD34 while attenuating the upregulation of GPA (Fig. 3D). Specifically, FA treatment caused a 5-fold increase in the frequency of cells coexpressing CD34 and GPA, two markers that minimally overlap during normal maturation (Fig. 3E). These results demonstrate cell-intrinsic defects in proliferation and maturation associated with aconitase inhibition in cultured erythroid progenitors.

### The effects of aconitase inhibition on erythropoiesis occur independently of metabolic impairment

**Figure 4:**
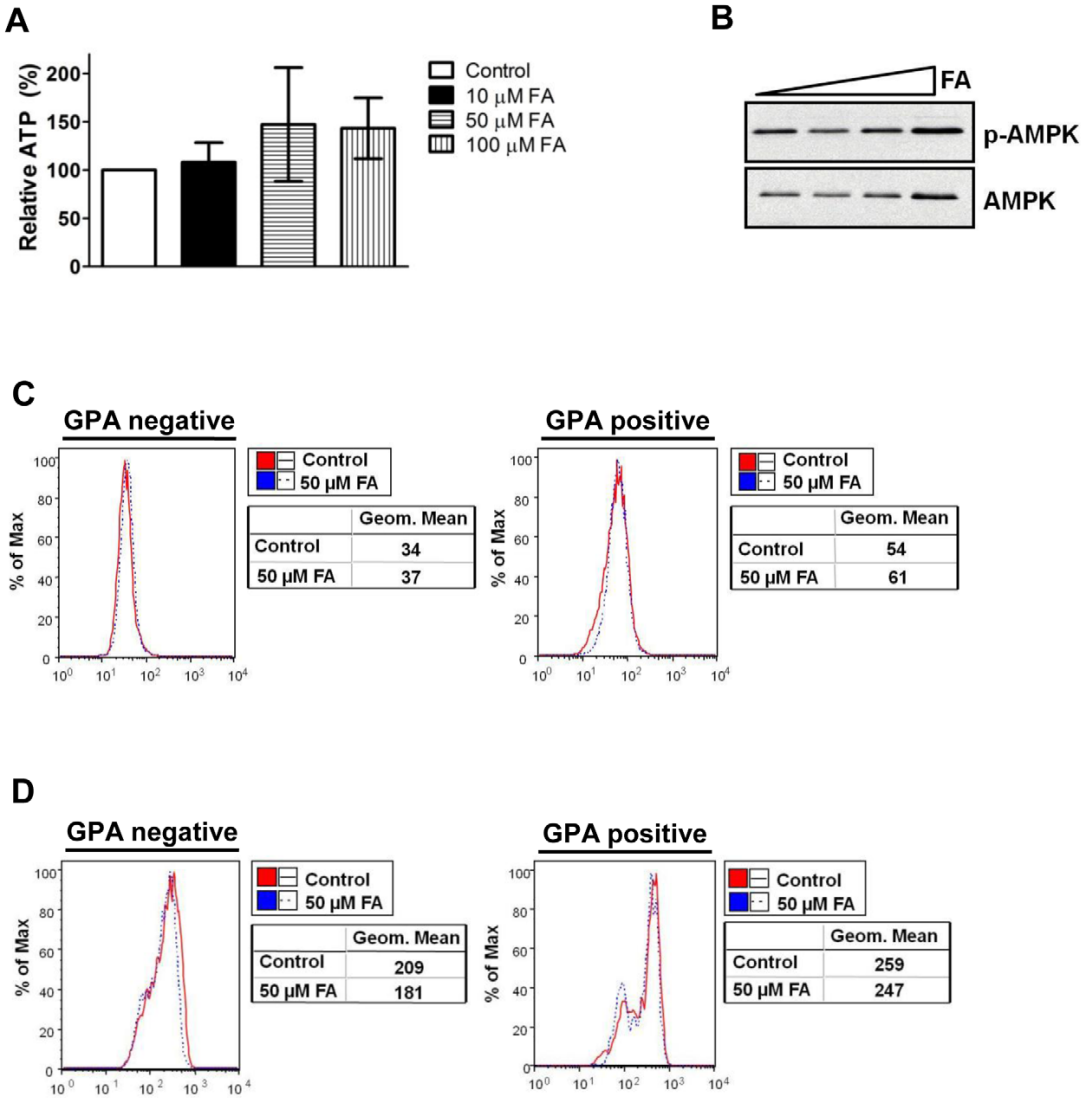
FA inhibits erythropoiesis in the absence of changes in cellular ATP, ROS, or MMP. (**A**) ATP levels in cells from day 5 erythroid cultures of human CD34^+^ progenitors treated with 0–100 µM FA. Shown are values relative to untreated cultures consisting of mean ± SD from three independent experiments. (**B**) APMK activation in cells from day 5 erythroid cultures with 0–100 µM FA assessed by immunoblotting of whole cell lysates for phospho- and total AMPK. (**C**) Levels of reactive oxygen species (ROS) in cells from day 3 erythroid cultures ±50 µM FA detected by the fluorescent probe APF. Mean fluorescence intensities (Geom. Mean) of the APF signal within GPA-negative and positive gates are shown adjacent to flow cytometry plots. (**D**) Mitochondrial membrane potential in cells from day 3 erythroid cultures ±50 µM FA detected by MitoTracker CMXRos. Mean fluorescence intensities (Geom. Mean) of the MitoTracker signal within GPA-negative and positive gates are shown adjacent to flow cytometry plots.

To determine whether the defect in erythropoiesis caused by aconitase inhibition occurred secondary to alterations in Krebs cycle metabolism or mitochondrial dysfunction, several parameters were assessed. Firstly, 50 µM FA had no effect on cellular steady state ATP levels in day 5 erythroid cultures (Fig. 4A). Secondly, FA treatment did not alter the activation status of AMP-kinase (AMPK) (Fig. 4B). AMPK functions as a sensor of the AMP∶ATP ratio; an increase in this ratio activates AMPK, which in turn upregulates catabolic pathways to restore cellular ATP levels [35]. Thirdly, the FA treatment did not alter levels of reactive oxygen species (ROS), as detected by the fluorescent indicator 3′-(aminophenyl)-fluorescein (APF) [36]. APF mean fluorescence intensity (MFI) was identical in untreated and FA-treated erythroid cultures, both in early (GPA negative gate) and late progenitors (GPA positive gate) (Fig. 4C). Fourthly, 50 µM FA did not alter the mitochondrial membrane potential (MMP), as assessed by staining with the membrane permeant dye MitoTracker CMXRos [37]. In both early (GPA negative) and more mature (GPA positive) populations, the MFI of MitoTracker staining was unaffected by FA treatment (Fig. 4D). These results demonstrate that the degree of aconitase inhibition sufficient to impair erythropoiesis is below the threshold that disrupts cellular ATP levels, redox status, or MMP.

### Aconitase modulates ERK signaling downstream of EPO

**Figure 5:**
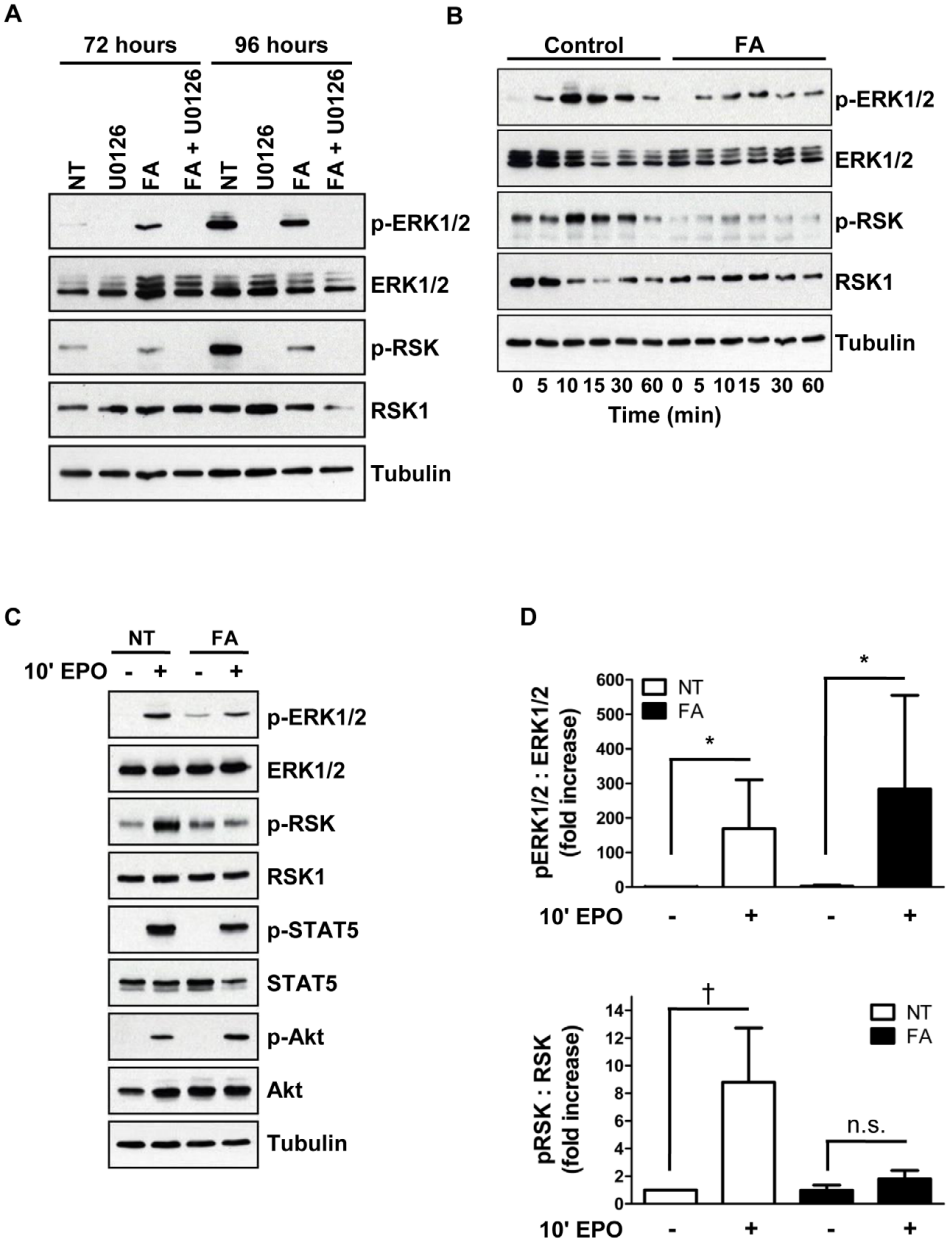
Aconitase inhibition interferes with EPO activation of ERK signaling in a pathway-specific manner. (**A**) Steady state phosphorylation of ERK and RSK as determined by immunoblotting of whole cell lysates of cells from days 3–4 erythroid cultures treated with 50 µM FA and 10 µM U0126 as indicated (NT, untreated). (**B**) Time course of ERK1/2 and RSK phosphorylation. Cells from day 3 erythroid cultures ±50 µM FA were subjected to 3 hours of cytokine starvation followed by stimulation with 4.5 U/ml EPO for the indicated time periods. (**C**) EPO induction of multiple downstream pathways in cells treated as in panel **B**. (**D**) Densitometric analysis of pERK1/2:ERK1/2 and pRSK:RSK levels in five independent experiments conducted as in panel **C**. Presented are mean values ± SEM for relative phosphoprotein signal divided by total protein signal; * *P*<0.05; ^†^ *P*<0.01; n.s., not significant.

Several studies have identified non-metabolic functions for aconitase, including participation in signaling modules [4], [7], [9]. Therefore experiments addressed whether aconitase inhibition affected the activation state of EPO-dependent signaling pathways. Immunoblotting with phospho-specific antibodies on whole cell lysates from erythroid cultures showed no effects of FA on basal signaling via JAK2-STAT5, calcineurin-NFAT, PI3K-Akt, PKC_α/βII_, and PLCγ pathways (data not shown) [38]–[42]. However, aconitase inhibition correlated with increased steady state levels of phosphorylated ERK (Fig. 5A). Unexpectedly, this increase in phospho-ERK was accompanied by a decrease in phosphorylation of p90 ribosomal S6 kinase (RSK) on an ERK target site, threonine 573 [43]. The requirement of MEK-ERK signaling for RSK T573 phosphorylation was confirmed by treatment of cells with the MEK inhibitor U0126 (Fig. 5A).

To characterize the effects of aconitase inhibition on the initiation of EPO signaling, erythroid progenitors pre-cultured with or without FA underwent cytokine starvation followed by acute stimulation with EPO. FA treatment did not prevent EPO activation of ERK but almost completely blocked its activation of RSK (Fig. 5B). In control cells treated with EPO, phospho-ERK levels peaked at 10 minutes, and phospho-RSK levels peaked at 15 minutes (Fig. 5B). In the FA treated cells, EPO induction of phospho-ERK also peaked at 10 minutes, but phospho-RSK showed no increase over the entire 1 hour time course. These signaling abnormalities were consistently observed in multiple independent experiments (Fig. 5D). By contrast, FA pretreatment had no effect on EPO induction of STAT5 or Akt activation (Fig. 5C). The data from steady state erythroid cultures and from EPO stimulation of cytokine-starved cells thus show that aconitase inhibition interferes with ERK-mediated activation of RSK.

### MEK inhibition recapitulates the effects of aconitase inhibition on erythropoiesis

**Figure 6:**
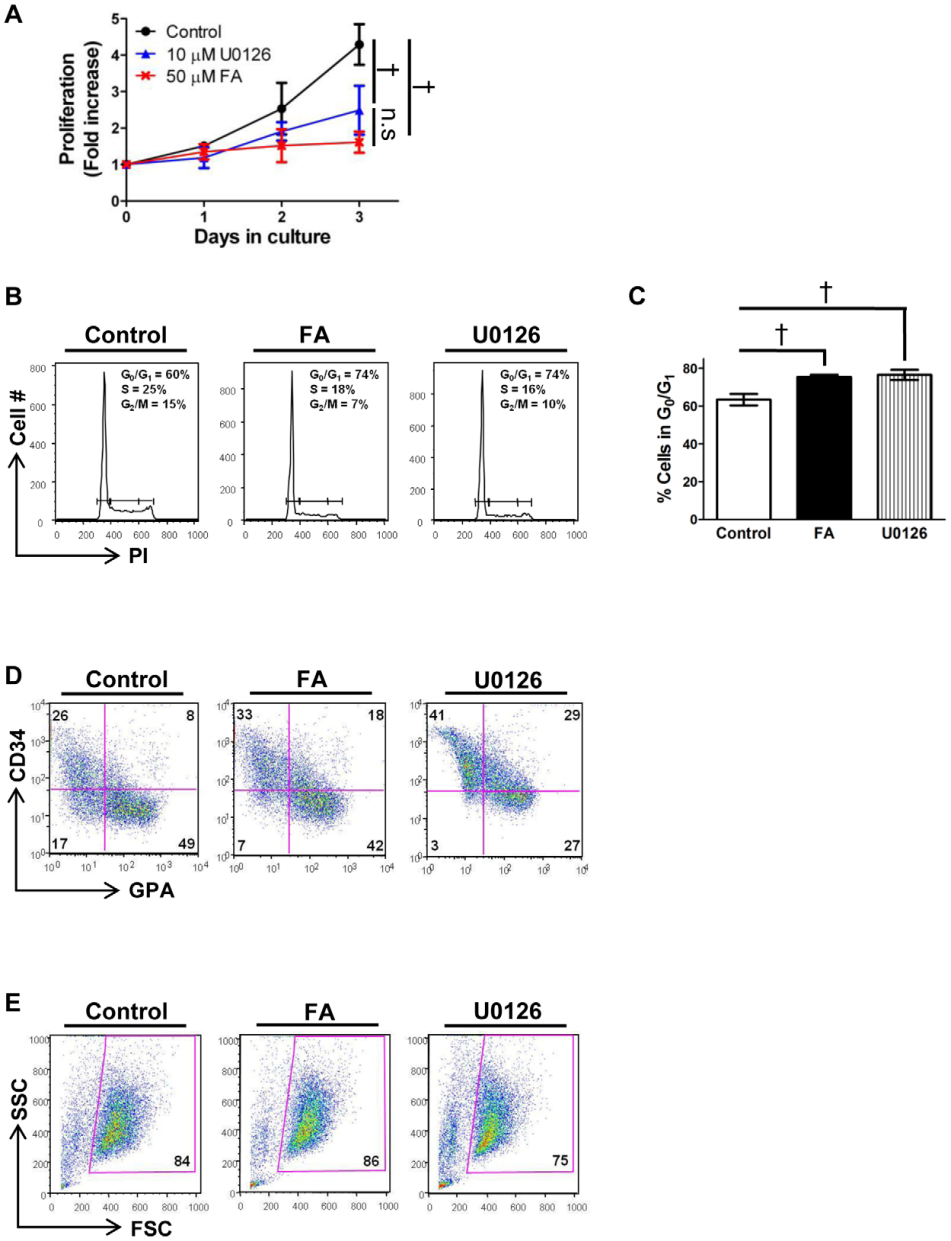
The effects of MEK inhibition on erythropoiesis recapitulate those of aconitase inhibition. (**A**) Fold increase in number of primary progenitors cultured for 3 days with either 50 µM FA or 10 µM U0126. Shown are mean ± SD for three independent experiments; ^†^ *P*<0.01. (**B**) Cell cycle profiles of cells from day 3 cultures as in panel **A** determined by propidium iodide staining followed by flow cytometry. Percentages of cells in G_0_/G_1_, S, and G_2_/M phases are indicated. (**C**) Multiple independent experiments as in panel **B** showing an increase in the percentage of cells in G_0_/G_1_ associated with either FA or U0126 treatment. Data represent mean ± SD; n = 3; ^†^ *P*<0.01. (**D**) Erythroid differentiation of progenitors cultured as in panel **A** assessed by staining for GPA and CD34 followed by flow cytometry; percentages of cells in each quadrant are shown. (**E**) Viable fractions from panel **D** determined by forward (FSC) and side (SSC) light scatter properties.

To determine whether impaired ERK signaling might underlie the erythroid defects associated with aconitase inhibition, FA and the MEK inhibitor U0126 were compared for their effects on erythropoiesis as in Figure 3. The MEK inhibitor at 10 µM reproducibly inhibited proliferation to the same degree as 50 µM FA (Fig. 6A), and both agents yielded similar cell cycle perturbations on propidium iodide staining, i.e. increased cells in G_0_/G_1_ (Figs. 6B,C). MEK inhibition also impaired erythroid differentiation in a manner similar to aconitase inhibition, characterized by the accumulation of an aberrant CD34^+^ GPA^+^ double positive population (Fig. 6D). As with aconitase inhibition, MEK inhibition minimally affected cell viability (Fig. 6E). The similar phenotypic effects resulting from FA and U0126 treatments thus support the notion that aconitase and ERK signaling participate in a common erythroid regulatory pathway.

### An aconitase-ERK protein complex that is disrupted by aconitase inhibition

**Figure 7:**
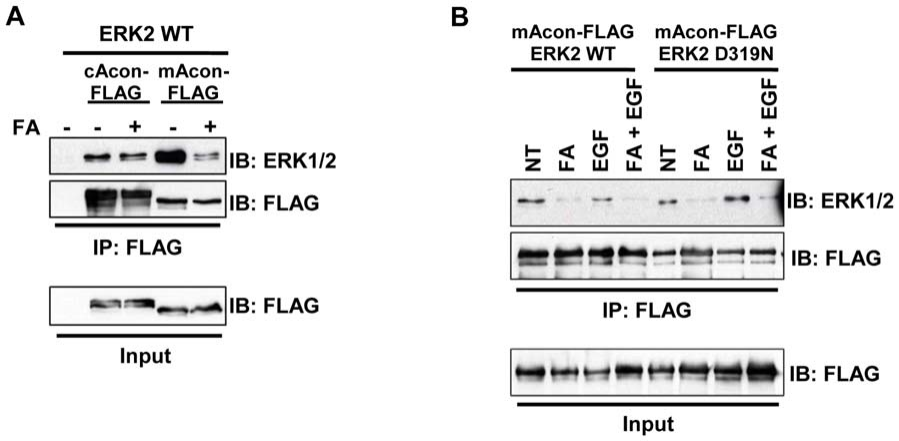
Identification of an ERK-aconitase protein complex that is disrupted by aconitase inhibition. (**A**) Assessment of ERK-aconitase interactions by coexpression followed by coimmunoprecipitation. ERK2 and FLAG-tagged cytosolic aconitase (cAcon-FLAG) or mitochondrial aconitase (mAcon-FLAG) were coexpressed in HEK293T cells ±500 µM FA. Immunoprecipitation (IP) with FLAG beads was followed by immunoblotting (IB) for total ERK and for the FLAG epitope. (**B**) Role of ERK D-domain interacting motif in ERK2 binding to mitochondrial aconitase. mAcon-FLAG was coexpressed with ERK2 WT or D319N in HEK293T cells ±500 µM FA, ±100 ng/ml EGF, followed by IP and IB as in panel **A**.

Large scale proteomic mapping of interaction networks in *Saccharomyces cerevisiae* has shown mitochondrial aconitase to interact with ∼20–30 partner proteins, of which one is KSS1, a yeast homolog of ERK; reciprocal analysis of KSS1 also identified aco1 (yeast mitochondrial aconitase) as an interaction partner [44]. Furthermore, high stringency scanning for motifs using Scansite 2.0 predicts ERK Docking Domains (D-Domains) in both aconitase isoforms [45]. To test for interaction between aconitase enzymes and ERK in a mammalian system, immunoprecipitation was conducted on epitope-tagged proteins co-expressed in HEK293T cells cultured under a variety of conditions, including FA and EGF treatment. Higher FA doses were used in these experiments due to the relative resistance of HEK293T cells to the growth inhibitory effects of FA, most likely related to high levels of endogenous aconitases. ERK bound both aconitase isoforms, showing a stronger interaction with mitochondrial aconitase (Fig. 7A). FA treatment potently and reproducibly diminished ERK2 interaction with mitochondrial aconitase. ERK activation by EGF treatment of serum-starved cells did not significantly affect the interaction, nor did mutation of the D-domain binding site in ERK2 (D319N) (Fig. 7B) [46]. ERK1 was also found to interact with mitochondrial aconitase (data not shown). Our data thus confirm an interaction of mitochondrial aconitase with ERK1/2, reinforcing previous findings in *Saccharomyces cerevisiae*. This interaction is disrupted by inhibition of aconitase activity but does not appear to depend on ERK activation or D-domain recognition.

Activation of cytoplasmic ERK targets such as RSK has been shown to depend on dimerization of ERK monomers [47]. Dimerization occurs in response to mitogenic stimuli, such as EGF in HEK293T cells, and provides a mechanism to spatially regulate ERK signaling. To test whether interaction with aconitase regulates ERK dimer formation, HEK293T cells ± FA and EGF were subjected to ERK2 dimer detection according to the method of Casar et al [47]. FA treatment had no effect on ERK2 dimer formation (Fig. S4), arguing against a role for aconitase in regulating ERK dimerization.

### Low dose aconitase inhibition normalizes red cell counts in a mouse model of polycythemia vera

**Figure 8:**
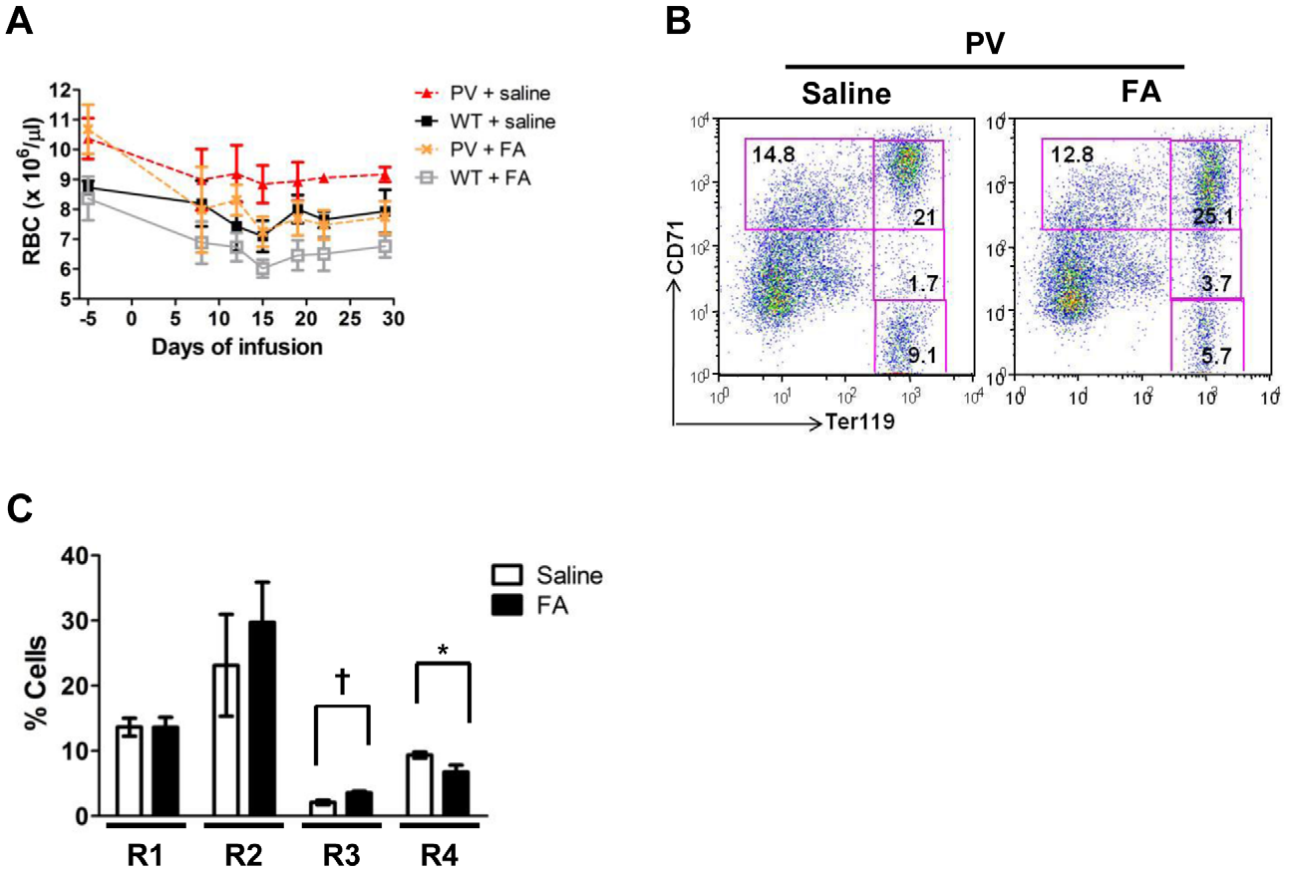
Aconitase inhibition normalizes red cell counts in a murine model of polycythemia vera. (**A**) RBC values over the course of a four-week infusion of saline (0.9%; n = 3) or FA (2 mg/kg/day; n = 10). Data represent mean ± SD. (**B**) Erythroid maturation in day 28 PV marrows. Boxes indicate erythroid developmental stages R1–R4. (**C**) Summary of data from panel **B** with mean percentages of cells in R1–R4 ± SD; n = 3 per group; ^*^ *P*<0.05; ^†^ *P*<0.01.

To determine whether aconitase inhibition could provide a therapeutic strategy for lowering red cell mass in polycythemia vera, we treated C57BL/6 mice expressing a human mutant *JAK2V617F* transgene (PV mice) and wild type (WT) littermate controls with a low dose infusion of FA (2 mg/kg/day) [48]. Mock-treated PV mice had significantly elevated red cell counts compared with normal controls (10.4–10.7×10^6^/µl in PV vs. 8.4–8.7×10^6^/µl in WT) (Fig. 8A). FA infusion normalized the RBC counts in PV mice after seven days and stably maintained these counts in the normal range for the duration of treatment (Fig. 8A). Platelet, neutrophil, and lymphocyte counts were not affected by FA treatment (Table S2). In PV marrows, as previously seen in WT marrows, FA increased the proportion of R3 erythroid precursors. A significant decrease in the percentage of more mature R4 precursors was also observed (Figs. 8B,C). These results suggest that a minor degree of aconitase inhibition in vivo may suffice to achieve clinically significant reductions in red cell mass in polycythemia vera.

To determine effects of aconitase inhibition on signaling by the constitutively active mutant JAK2V617F, Ba/F3 transfectants expressing EPOR ± JAK2V617F [49], [50] underwent treatment with FA. These cells however were resistant to growth inhibition by FA doses 20-fold higher than those effective on primary erythroid progenitors (Fig. S5). As an additional complication, the Ba/F3 EPOR JAK2V617F cells showed a paradoxical response to EPO stimulation consisting of marked downregulation of RSK phosphorylation (Fig. S6).

## Discussion

Inactivation of aconitase enzymes contributes to the erythroid response to iron restriction [4]. However, because iron deprivation simultaneously affects multiple signaling and metabolic pathways, the exact role of aconitases in erythropoietic regulation has remained unclear. The current studies have directly examined the role of aconitases during erythroid development by use of a specific active site inhibitor. Application of this approach has confirmed that aconitase activity potently regulates erythropoiesis in vivo and in vitro. In vivo, aconitase inhibition caused a rapid-onset hypoplastic anemia with slightly macrocytic, normochromic red cells (Table 1). These red cell features suggest that the anemia did not arise from impaired production of heme due to deficiency of succinyl CoA. Anemias caused by defective heme synthesis, such as those due to deficiencies of erythroid-specific δ-aminolevulinate synthase or ferrochelatase, are characteristically microcytic and hypochromic [51], [52]. The low reticulocyte counts combined with increased serum EPO levels (Figs. 1A,B) suggest that FA treatment interfered with red cell production, a conclusion further supported by the poor reticulocyte response to phenylhydrazine challenge seen in FA-treated animals (Fig. 2B). Furthermore, in vivo and in vitro studies both showed impaired differentiation of erythroid progenitors associated with aconitase inhibition (Figs. 1C, 3D, 8B).

Precise definition of the isoform-specific contributions of aconitases to erythroid regulation will require development of new conditional knockout mice with individual and compound gene deletions. Several lines of evidence, however, suggest that mitochondrial aconitase plays a key role. Firstly, fluoroacetate acts predominantly as an inhibitor of mitochondrial aconitase, based on the location of its enzymatic conversion to fluorocitrate, the active inhibitor [53]. Secondly, infusion of FA in mice lacking cytosolic aconitase (IRP1−/−) induced an anemia identical to that occurring in wild-type mice (Fig. S7). Thirdly, IRP1−/− mice showed no hematologic defects in response to anemic challenge (Figs. 2C,D). Fourthly, mitochondrial aconitase bound ERK more avidly than cytosolic aconitase and, unlike its cytosolic counterpart, displayed modulation of ERK binding by FA (Fig. 7). Several studies have previously demonstrated mitochondrial regulation of erythroid differentiation and proliferation. For example, the inhibition of mitochondrial biogenesis by chloramphenicol is associated with impaired erythropoiesis in vivo and in vitro [54], [55]. The ability of exogenous isocitrate to reverse in vitro differentiation defects caused by chloramphenicol has suggested that mitochondrial aconitase may participate in the regulation of erythropoiesis by mitochondrial function [4]. Further supporting this notion, multiple murine models of impaired mitochondrial function all show a similar, distinctive erythroid maturation defect, i.e. an increase in CD71^Intermediate^ Ter119^Bright^ progenitors (R3 stage), closely resembling the defect seen with FA infusion (Figs. 1C and 8B) [56]–[58].

Among the various pathways downstream of EPO regulating erythroid development, only the ERK pathway displayed consistent modulation in response to aconitase inhibition. In particular, FA treatment diminished RSK T573 phosphorylation in erythroid progenitors subjected to either acute or chronic EPO stimulation (Fig. 5). The effects of aconitase inhibition on ERK signaling are likely to be biologically relevant because direct inhibition of ERK activation with the MEK inhibitor U0126 recapitulated several key features of the erythroid response to FA (Fig. 6). By contrast, our data suggest that the effects of FA were unrelated to changes in ATP, ROS, or mitochondrial membrane potential (Fig. 4). Why FA had no effect on these latter parameters may be related to the dose employed, to possible metabolic compensation by cytosolic aconitase, or to the dependence of early hematopoietic progenitors more on glycolysis than on oxidative phosphorylation.

The mechanism underlying the cross-talk between aconitase and ERK, while unclear, may relate to the assembly of protein complexes containing ERK and mitochondrial aconitase (Fig. 7). Interestingly, such complexes are phylogenetically conserved back to yeast and are regulated by aconitase activity. Previous studies have documented localization of ERK in mitochondria [20], [59], [60]. Whether mitochondrial aconitase participates in an organelle-specific ERK scaffolding complex, as has been described for endosomes and Golgi [17], [18], remains a subject of investigation. Nevertheless, the current studies provide a novel function for aconitase as a licensing factor for erythropoietin-dependent transduction of ERK signaling. This regulatory input could permit adjustment of ERK activity in accordance with aconitase function, which in turn is affected by iron availability, redox, and iron-sulfur cluster assembly pathways. In this manner, a variety of signals reflecting mitochondrial status potentially could be transmitted to the machinery driving proliferation and differentiation.

The ability to modulate red cell production with low dose aconitase inhibition may have therapeutic implications for polycythemia vera. Phlebotomy, the primary treatment for this disease, controls red cell mass via induction of iron deficiency, most likely acting through erythroid inactivation of aconitase. Attainment of iron deficiency may require numerous blood draws over a prolonged period and may be complicated by significant thrombocytosis [61]. Repeated phlebotomy also poses a risk for phlebitis and infection. In a mouse model of JAK2 V617F-driven polycythemia, low dose aconitase inhibition rapidly normalized red cell counts in a lineage specific manner without causing anemia (Fig. 8, Table S2). However, further studies are needed to determine the clinical feasibility of this approach. Fluoroacetate is a known toxic agent that targets the cardiovascular and central nervous systems in mice and humans. Reported lethal doses range from 0.5 to 19 mg/kg in mice and 2 to 10 mg/kg in humans [62]. While a continuous FA infusion of 2 mg/kg/day corrects red cell counts in PV mice in the absence of overt symptoms, toxicology studies will be necessary to rule out adverse effects of both acute and chronic administration.

## Materials and Methods

### Mice

PV mice consisted of line A JAK2V617F transgenic mice between the ages of 10 and 14 weeks [48]. IRP1−/− mice were generously donated by Dr. Tracey Rouault (National Institute of Child Health & Human Development, Bethesda, MD) [32]. Animal studies were approved by the University of Virginia Animal Care and Use Committee (ACUC, protocol 3545, latest annual renewal approved on 6.15.10). Mice were housed in a pathogen-free facility and handled in accordance with the policies of the University of Virginia ACUC. For continuous in vivo infusions, a sterile 0.9% saline solution or sodium fluoroacetate (FA) solution prepared in 0.9% saline was delivered subcutaneously using Alzet osmotic pumps model 1002 or 1004 (Durect, Cupertino, CA) based on the approach of Isken and colleagues [28]. Phenylhydrazine (Sigma, St. Louis, MO) prepared in sterile phosphate-buffered saline (PBS) was administered by intraperitoneal bolus injection at doses of 30 mg/kg/day×2 days (FA-treated mice) or 60 mg/kg/day×2 days (saline-treated mice). Blood was collected from the retro-orbital plexus into EDTA-coated micro tubes (BD Biosciences, Franklin Lakes, NJ) using heparinized micro-capillary tubes. Complete blood counts were measured on a Hemavet 850FS automated analyzer (Drew Scientific, Dallas, TX). Serum EPO levels were determined with the Quantikine Mouse EPO ELISA kit (R&D Systems, Minneapolis, MN).

### Cell culture

Peripheral blood mobilized, purified human CD34^+^ cells were obtained from the Hematopoietic Cell Processing Core at the Fred Hutchinson Cancer Center (Seattle, WA) as previously described [4]. After 72 hours in multi-lineage serum-free medium, the cells were seeded in serum-free erythroid differentiation medium consisting of Iscove's modified Dulbecco medium (IMDM) supplemented with 4.5 U/ml EPO (Amgen, Thousand Oaks, CA), 25 ng/ml stem cell factor (PeproTech, Rocky Hill, NJ), bovine serum albumin (BSA, Sigma), and 100% Fe-saturated transferrin (Stem Cell Technologies, Vancouver, Canada) [4]. Sodium fluoroacetate was purchased from Sigma, and U0126 from Cell Signaling Technology (Beverly, MA). For cytokine starvation experiments, cells that had been cultured in erythroid medium for 72 hours were washed in PBS and re-suspended in IMDM supplemented with 0.1% BSA for 3 hours, followed by stimulation with 4.5 U/ml EPO. Murine Ba/F3 EPOR and Ba/F3 EPOR JAK2V617F cell lines have been previously described [63]. Both cells lines were maintained in Roswell Park Memorial Institute (RPMI) medium supplemented with 10% fetal bovine serum (Invitrogen, Carlsbad, CA) and 2 ng/ml murine IL-3 (PreproTech). For cytokine starvation experiments, cells were washed twice in RPMI and cultured for 4 hours in RPMI prior to stimulation with 10 U/ml EPO (Amgen).

### Plasmids and transfections

pcDNA3-HA-ERK2 wt and D319N, as well as pcDNA3-T7-ERK1, were purchased from Addgene (Cambridge, MA). Full-length cDNAs for human *ACO1* (encoding cytosolic aconitase) and *ACO2* (encoding mitochondrial aconitase) (Open Biosystems, Huntsville, AL) were subcloned into the *Xho*I and *Not*I sites of pCMV Tag4A (Stratagene, La Jolla, CA) to express mAcon-FLAG and cAcon-FLAG. A 3′ *Sal*I, rather than *Xho*I, site was introduced into the *ACO2* insert. Transient transfections in HEK293T cells were carried out using the calcium phosphate method, as previously described [64]. Cells were serum-started for 6 hours, followed by stimulation with 100 ng/ml epidermal growth factor (EGF, PeproTech) for 5 minutes prior to harvest where indicated. 36 hours post-transfection, cells were resuspended in NP-40 extraction buffer containing 50 mM Tris-HCl (pH 7.6), 150 mM NaCl, 0.5% NP-40, 1 mM MgCl_2_, 50 µM ZnSO_4_, EDTA-free protease inhibitor cocktail (Roche, Indianapolis, IN), 1 mM sodium orthovanadate and 10 mM sodium fluoride. Following a 20 minute incubation at 4°C, insoluble debris was removed by centrifugation.

### Immunoblot and immunoprecipitation

Western blot analysis was performed as previously described after sodium dodecyl sulfate-poly-acrylamide gel electrophoresis (SDS-PAGE) [65]. Antibodies to phospho-ERK1/2 (T202/Y204), ERK1/2, RSK1, phospho-STAT5 (Y694), STAT5, phospho-Akt (S473), Akt, AMPK, and phospho-AMPK (T172) were purchased from Cell Signaling Technology. Anti-phospho-RSK (T573) was purchased from Epitomics (Burlingame, CA); anti-caspase 3 from Santa Cruz Biotechnology (Santa Cruz, CA). Anti-tubulin and anti-actin were purchased from Sigma. Immunoprecipitation of aconitase-containing complexes was performed by incubating cellular extracts with M2 agarose FLAG beads (Sigma) for 2 hours at 4°C. Following washing of beads, proteins eluted in SDS-PAGE loading buffer underwent SDS-PAGE and immunoblot analysis using antibodies to ERK1/2 (Cell Signaling Technology) and FLAG (Sigma). Densitometry data were acquired on a GS800 calibrated densitometer (Bio-Rad, Hercules, CA) and analyzed with the Quantity One software (Bio-Rad). For ERK2 dimer formation analysis, HEK293T cells were serum-starved overnight ±500 µM FA and stimulated with 100 ng/ml EGF for 5 minutes prior to harvest and analysis by native gel electrophoresis as described [66].

### Flow cytometry

Cells were analyzed on a FACSCalibur instrument (Becton Dickinson, San Jose, CA). All fluorochrome-conjugated antibodies were purchased from BD Pharmingen (San Diego, CA), with the exception of PE-anti-CD71 (Dako, Carpinteria, CA). Erythroid differentiation was assessed by co-staining human erythroid progenitors with fluorochrome-conjugated antibodies to GPA (FITC or PE), CD71 (PE), CD34 (APC), and CD44 (FITC). Reticulocytes were measured by staining whole blood with thiazole orange as described [67]. Bone marrows were harvested from femurs into PBS supplemented with 5 mM EDTA and stained with APC-anti-Ter119 and PE-anti-CD71. Cell death was analyzed with the Annexin V-PE Apoptosis Detection Kit I (BD Pharmingen). For cell cycle analysis, cells underwent ethanol fixation and RNase A treatment followed by propidium iodide staining. Data was analyzed using the FlowJo software (version 8.6.3, TreeStar Inc., Ashland, OR).

### Metabolic assays

Citrate measurements were performed using the BioVision Citrate Assay kit according to the manufacturer's instructions (BioVision, Mountain View, CA). To measure intracellular levels, metabolic extracts from day 5 untreated and 50 µM FA-treated CD34^+^ erythroid cultures were obtained as previously described [4]. Fluorimetry was performed on a FluoSTAR Optima fluorescent microplate reader (BMG Labtech, Cary, NC) and analyzed with the MARS data analysis software (version 1.01, BMG Labtech). To measure serum citrate, whole blood was collected retro-orbitally and allowed to clot at room temperature for 30 minutes. Clotted samples were centrifuged at 13,000 g for 5 minutes at room temperature and sera were stored at −20°C. Samples were deproteinized using the Deproteinization Sample Preparation kit (BioVision) according to the manufacturer's instructions.

ATP measurements were performed as described [4]. Staining of cells with 3′-(aminophenyl)-fluorescein (APF) followed the manufacturer's guidelines (Invitrogen). Briefly, CD34 cells ±50 µM FA were stained with APC-anti-GPA (BD Pharmingen) and APF, and mean fluorescence intensity of the APF dye was determined by flow cytometry after gating on GPA-negative and positive populations. Mitochondrial membrane potential was determined by staining cells with MitoTracker Red CMXRos dye (Invitrogen) according to the manufacturer's instructions, followed by staining for GPA. The mean fluorescence intensity of MitoTracker stained cells was determined by flow cytometry after gating on GPA-negative and positive populations.

### Statistical analysis

GraphPad Prism software version 5.01 (GraphPad Software, Inc., La Jolla, CA) was used to display the data graphically and to perform statistical analysis. Results were analyzed by unpaired two-tailed Student's *t* test or one-way analysis of variance (ANOVA) with Newman-Keuls post-test when comparing multiple groups. *P* values less than or equal to 0.05 were considered significant.

## Supporting Information

Figure S1 **Elevated serum citrate levels in mice treated with fluoroacetate.** Sera from saline-treated and FA-treated (2 mg/kg/day) mice were isolated from whole blood collected on day 26 post initiation of treatment. Citrate levels were determined enzymatically. Data are presented as mean ± SD; n = 8 per group; ^‡^ *P*<0.001.
(TIF)

Figure S2 **Analysis of the bone marrow erythroid maturation using Ter119 and CD44.** (**A**) Erythroid maturation in marrows from mice who received a four-week infusion of FA (2 mg/kg/day) or saline (0.9%). After gating on the Ter119^+^ fraction, cells were analyzed for CD44 expression versus Forward Scatter (FSC). Representative flow cytometry plots are shown. Roman numerals I-V indicate distinct erythroid subpopulations. (**B**) Summary of data from panel **A** with mean percentages of cells in subpopulations I-V ± SD; n = 4 per group; ^*^ *P*<0.05. (**C**) Summary of data from panel **A** with absolute number of cells per femur in subpopulations I-V ± SD; n = 4 per group; ^†^ *P*<0.01.
(TIF)

Figure S3 **Increased intracellular citrate in FA-treated cells.** CD34^+^ cells were cultured in erythroid medium for 5 days±50 µM FA. Cellular extracts were assayed for citrate levels enzymatically. Data are presented as mean fold increase over control cells ± SD; n = 4; ^†^ *P*<0.01.
(TIF)

Figure S4 **Aconitase inhibition does not prevent ERK dimerization.** Detection of ERK2 dimer formation upon EGF stimulation by native gel electrophoresis followed by immunoblotting. HEK293T cells ±500 µM FA, ±10 µM U0126 were cytokine-starved overnight and stimulated with 100 ng/ml EGF for 5 minutes prior to harvest.
(TIF)

Figure S5 **Ba/F3 cells expressing EPOR or EPOR plus JAK2V617F are relatively resistant to the growth inhibitory effects of FA.** (**A**) Proliferation of Ba/F3 EPOR cells cultured in the presence of 2 ng/ml murine IL-3 and treated with 0–5 mM FA for 72 hours. Data are presented as mean ± SD; n = 3; * *P*<0.05; n.s., not significant. (**B**) Proliferation of Ba/F3 EPOR JAK2V617F cells cultured as in panel **A**. Data are presented as mean ± SD; n = 3; ^†^ *P*<0.01.
(TIF)

Figure S6 **Paradoxical RSK deactivation in Ba/F3 EPOR JAK2V617F cells in response to EPO stimulation.** Ba/F3 EPOR and Ba/F3 EPOR JAK2V617F cells were starved of cytokines for four hours prior to stimulation with 10 U/ml EPO for ten minutes. Phosphorylation of ERK and RSK was assessed by immunoblotting of whole cell lysates.
(TIF)

Figure S7 **Mice lacking cytosolic aconitase respond normally to fluoroacetate infusion.** (**A**) RBC and hematocrit values in WT, IRP1+/− and IRP1−/− mice treated for 23 days with (2 mg/kg/day). Data represent mean ± SD; n = 6 per group. (**B**) Absolute reticulocyte counts in the WT, IRP1-/WT and IRP1−/− groups, as in panel **A**. (**C**) Erythroid maturation in marrows from FA-treated animals. Representative flow cytometry plots are shown. Boxes indicate erythroid developmental stages R1–R4. (**D**) Summary of results from panel **C** showing mean percentages of cells in R1–R4 ± SD; n = 3 per group.
(TIF)

Table S1 CBC parameters of WT mice treated for four weeks with saline (0.9%) or FA (2 mg/kg/day).
(DOC)

Table S2 CBC parameters of PV mice treated for four weeks with saline (0.9%) or FA (2 mg/kg/day).
(DOC)
