# Supplementary figures and images for: Aconitase Regulation of Erythropoiesis Correlates with a Novel Licensing Function in Erythropoietin-Induced ERK Signaling
Source: PLoS One. 2011 Aug 22;6(8):e23850. doi: 10.1371/journal.pone.0023850 (PMC3161794; doi:10.1371/journal.pone.0023850)

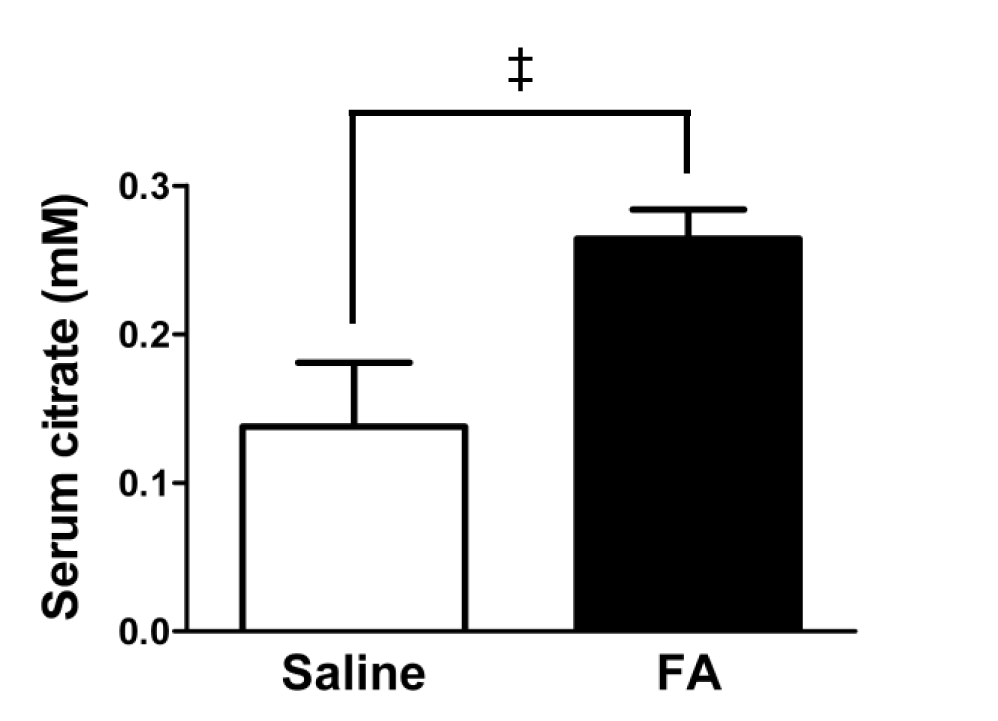

Supplement: Figure S1 — Elevated serum citrate levels in mice treated with fluoroacetate. Sera from saline-treated and FA-treated (2 mg/kg/day) mice were isolated from whole blood collected on day 26 post initiation of treatment. Citrate levels were determined enzymatically. Data are presented as mean ± SD; n = 8 per group; ‡ P<0.001. (TIF) [file pone.0023850.s001.tif]

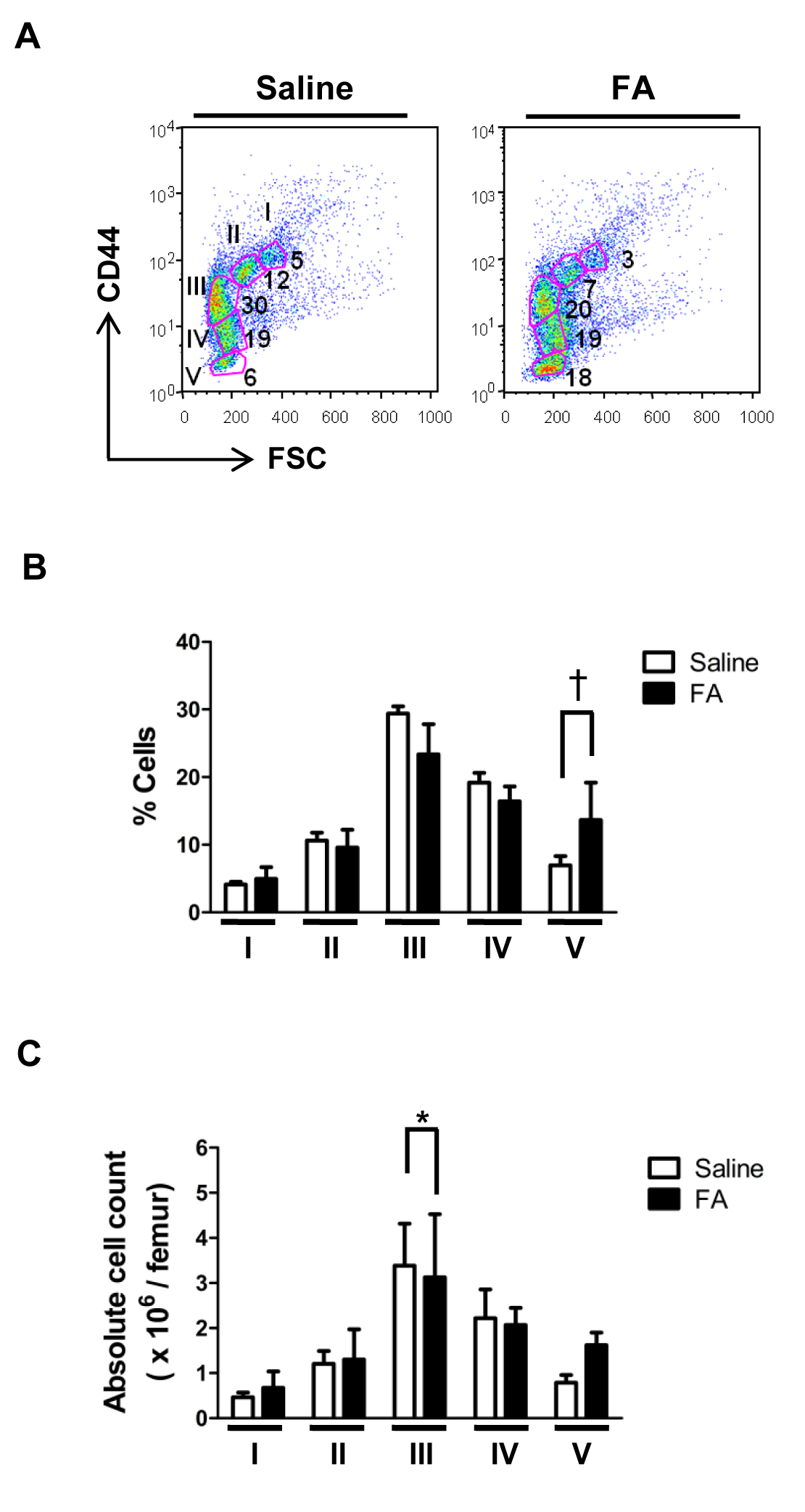

Supplement: Figure S2 — Analysis of the bone marrow erythroid maturation using Ter119 and CD44. (A) Erythroid maturation in marrows from mice who received a four-week infusion of FA (2 mg/kg/day) or saline (0.9%). After gating on the Ter119+ fraction, cells were analyzed for CD44 expression versus Forward Scatter (FSC). Representative flow cytometry plots are shown. Roman numerals I-V indicate distinct erythroid subpopulations. (B) Summary of data from panel A with mean percentages of cells in subpopulations I-V ± SD; n = 4 per group; * P<0.05. (C) Summary of data from panel A with absolute number of cells per femur in subpopulations I-V ± SD; n = 4 per group; † P<0.01. (TIF) [file pone.0023850.s002.tif]

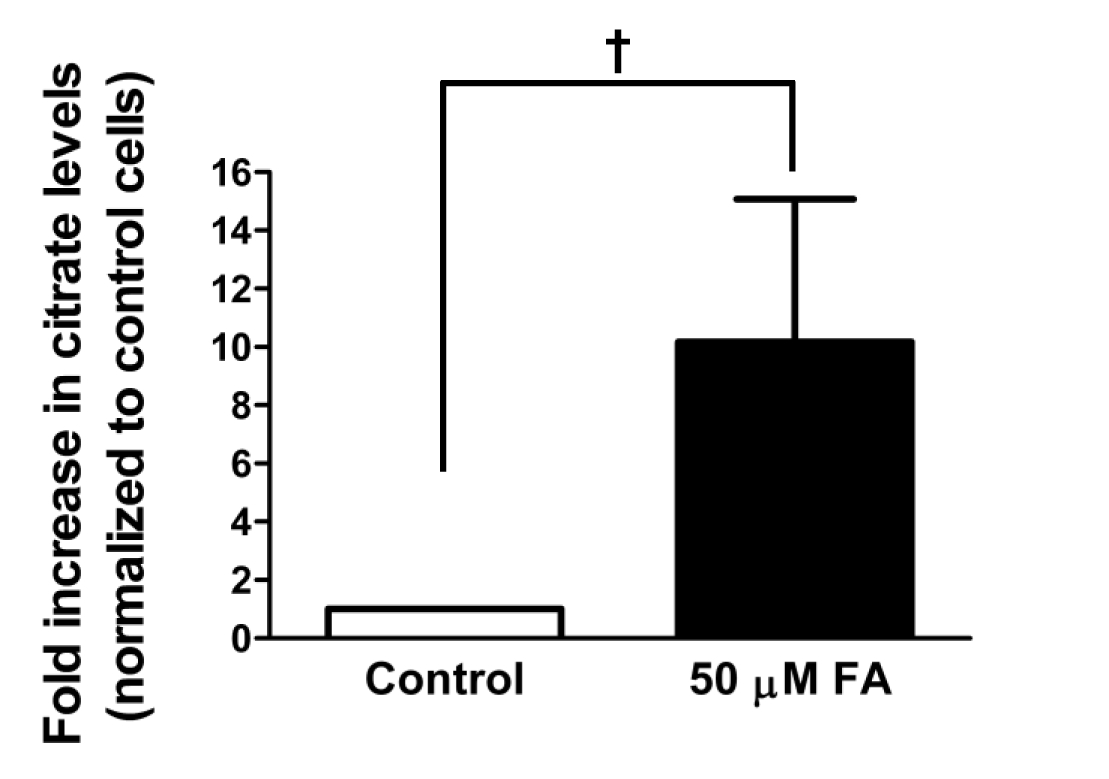

Supplement: Figure S3 — Increased intracellular citrate in FA-treated cells. CD34+ cells were cultured in erythroid medium for 5 days±50 µM FA. Cellular extracts were assayed for citrate levels enzymatically. Data are presented as mean fold increase over control cells ± SD; n = 4; † P<0.01. (TIF) [file pone.0023850.s003.tif]

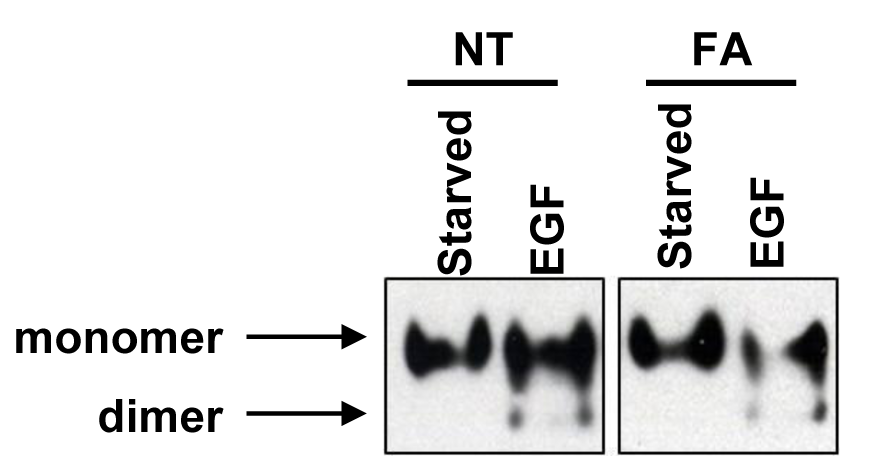

Supplement: Figure S4 — Aconitase inhibition does not prevent ERK dimerization. Detection of ERK2 dimer formation upon EGF stimulation by native gel electrophoresis followed by immunoblotting. HEK293T cells ±500 µM FA, ±10 µM U0126 were cytokine-starved overnight and stimulated with 100 ng/ml EGF for 5 minutes prior to harvest. (TIF) [file pone.0023850.s004.tif]

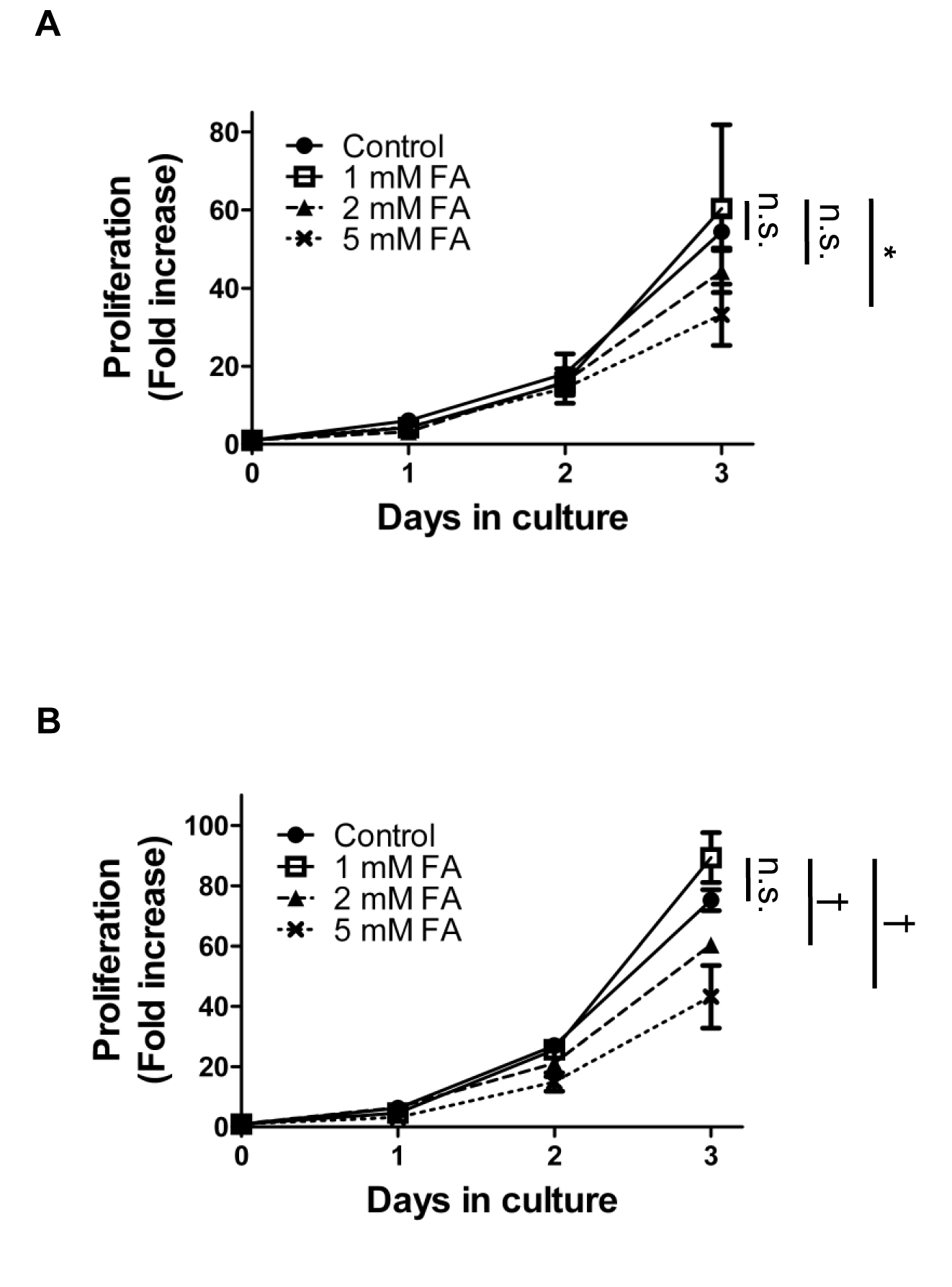

Supplement: Figure S5 — Ba/F3 cells expressing EPOR or EPOR plus JAK2V617F are relatively resistant to the growth inhibitory effects of FA. (A) Proliferation of Ba/F3 EPOR cells cultured in the presence of 2 ng/ml murine IL-3 and treated with 0–5 mM FA for 72 hours. Data are presented as mean ± SD; n = 3; * P<0.05; n.s., not significant. (B) Proliferation of Ba/F3 EPOR JAK2V617F cells cultured as in panel A. Data are presented as mean ± SD; n = 3; † P<0.01. (TIF) [file pone.0023850.s005.tif]

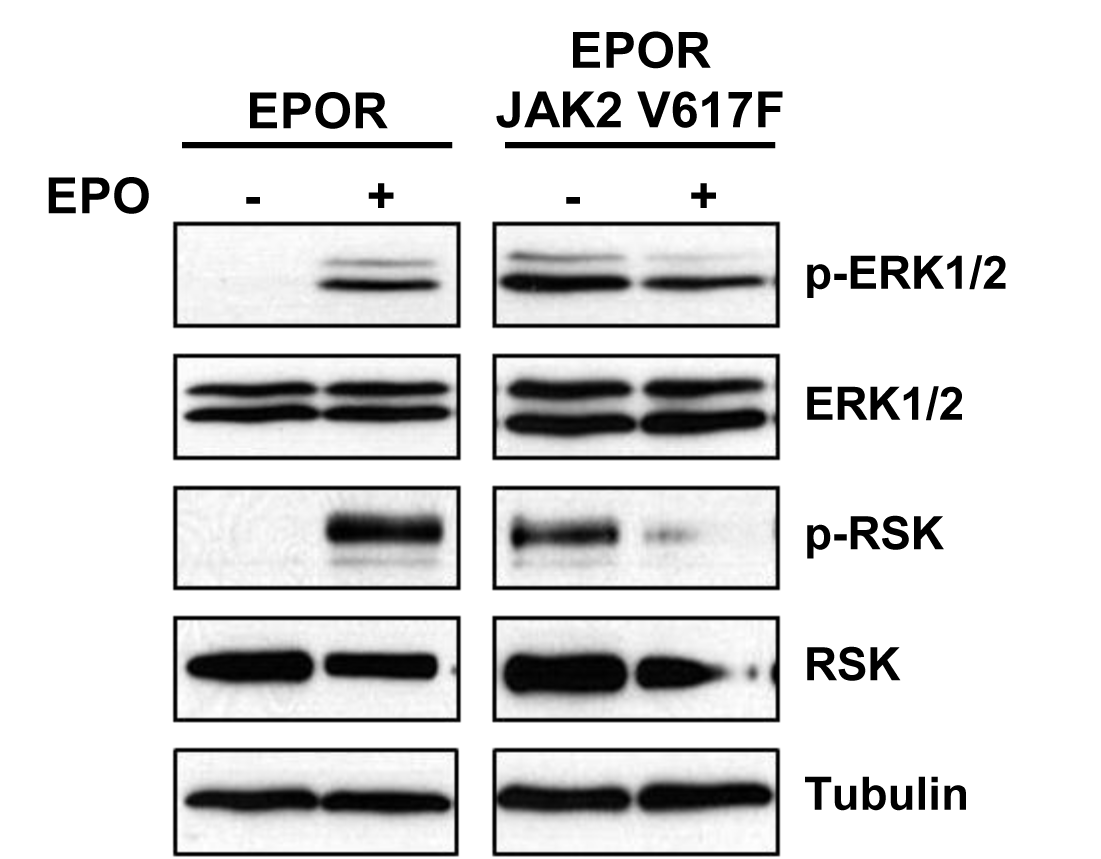

Supplement: Figure S6 — Paradoxical RSK deactivation in Ba/F3 EPOR JAK2V617F cells in response to EPO stimulation. Ba/F3 EPOR and Ba/F3 EPOR JAK2V617F cells were starved of cytokines for four hours prior to stimulation with 10 U/ml EPO for ten minutes. Phosphorylation of ERK and RSK was assessed by immunoblotting of whole cell lysates. (TIF) [file pone.0023850.s006.tif]

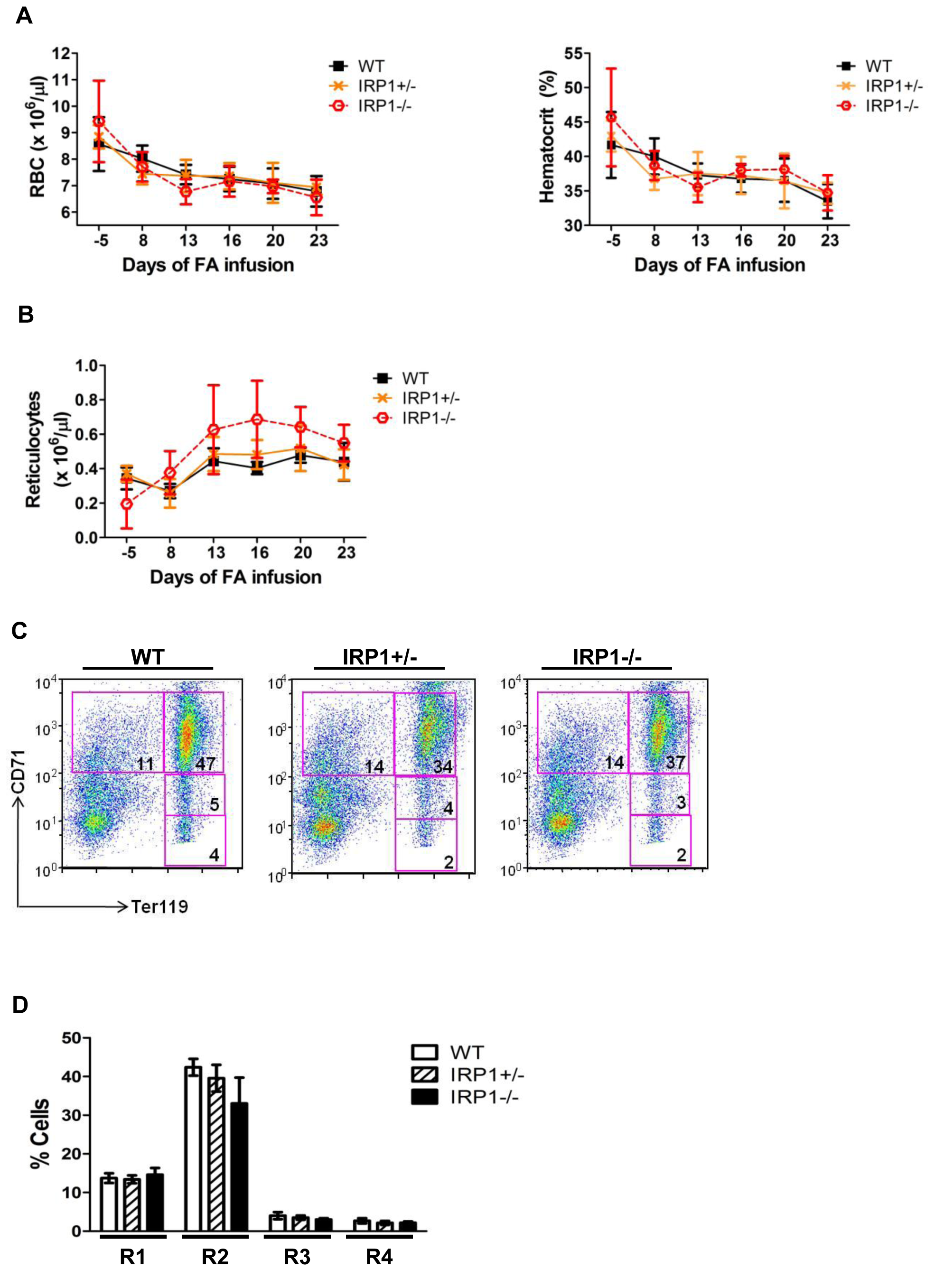

Supplement: Figure S7 — Mice lacking cytosolic aconitase respond normally to fluoroacetate infusion. (A) RBC and hematocrit values in WT, IRP1+/− and IRP1−/− mice treated for 23 days with (2 mg/kg/day). Data represent mean ± SD; n = 6 per group. (B) Absolute reticulocyte counts in the WT, IRP1-/WT and IRP1−/− groups, as in panel A. (C) Erythroid maturation in marrows from FA-treated animals. Representative flow cytometry plots are shown. Boxes indicate erythroid developmental stages R1–R4. (D) Summary of results from panel C showing mean percentages of cells in R1–R4 ± SD; n = 3 per group. (TIF) [file pone.0023850.s007.tif]
