# Supplementary material for: Aconitase Regulation of Erythropoiesis Correlates with a Novel Licensing Function in Erythropoietin-Induced ERK Signaling
Source: PLoS One. 2011 Aug 22;6(8):e23850. doi: 10.1371/journal.pone.0023850 (PMC3161794; doi:10.1371/journal.pone.0023850)
Supplement: Table S1 — CBC parameters of WT mice treated for four weeks with saline (0.9%) or FA (2 mg/kg/day). (DOC) [file pone.0023850.s008.doc]

**Table S1.**  CBC parameters of WT mice treated for four weeks with saline (0.9%) or FA (2 mg/kg/day).

| **Blood parameter** | **Saline**  **(n = 3)** | **FA-treated**  **(n = 10)** | ***P***  **value** |
| --- | --- | --- | --- |
| **RBC x 10^6^/μL** | 7.93 ± 0.72 | 6.76 ± 0.37 | 0.0024* |
| **Hematocrit, %** | 35.60 ± 3.64 | 34.62 ± 1.39 | 0.47 |
| **Hemoglobin, g/dL** | 12.20 ± 1.56 | 11.76 ± 1.45 | 0.18 |
| **MCHC, g/dL** | 34.57 ± 4.77 | 33.98 ± 1.13 | 0.70 |
| **MCV, fL** | 44.83 ± 0.6 | 51.16 ± 1.24 | <0.0001* |
| **Platelets, x 10^3^/μL** | 866 ± 46 | 866 ± 147.6 | 0.99 |
| **Neutrophils, x 10^3^/μL** | 1.78 ± 1.00 | 2.34 ± 1.13 | 0.43 |
| **Lymphocytes, x 10^3^/μL** | 5.50 ± 2.04 | 5.68 ± 0.77 | 0.91 |

Data are presented as mean ± SD.

^*^ indicate values that are significantly different from saline-

treated mice.
