# Supplementary material for: Aconitase Regulation of Erythropoiesis Correlates with a Novel Licensing Function in Erythropoietin-Induced ERK Signaling
Source: PLoS One. 2011 Aug 22;6(8):e23850. doi: 10.1371/journal.pone.0023850 (PMC3161794; doi:10.1371/journal.pone.0023850)
Supplement: Table S2 — CBC parameters of PV mice treated for four weeks with saline (0.9%) or FA (2 mg/kg/day). (DOC) [file pone.0023850.s009.doc]

**Table S2.**  CBC parameters of PV mice treated for four weeks with saline (0.9%) or FA (2 mg/kg/day).

| **Blood parameter** | **Saline**  **(n = 3)** | **FA-treated**  **(n = 10)** | ***P***  **value** |
| --- | --- | --- | --- |
| **RBC, x 10^6^/μL** | 9.16 ± 0.25 | 7.73 ± 0.54 | .002* |
| **Hematocrit, %** | 38.20 ± 2.20 | 35.41 ± 1.83 | .037* |
| **Hemoglobin, g/dL** | 13.53 ± 0.57 | 12.59 ± 0.72 | .068 |
| **MCHC, g/dL** | 35.47 ± 0.67 | 35.93 ± 3.0 | .8 |
| **MCV, fL** | 40.2 ± 1.04 | 45.52 ± 1.56 | .0003* |
| **Platelets, x 10^3^/μL** | 1841 ± 672 | 1785 ± 416 | .86 |
| **Neutrophils, x 10^3^/μL** | 1.43 ± 0.41 | 3.91 ± 2.42 | .25 |
| **Lymphocytes, x 10^3^/μL** | 5.11 ± 0.17 | 6.97 ± 3.17 | .35 |

Data are presented as mean ± SD.

^*^ indicate values that are significantly different from saline-

treated mice.
